# The biocontrol agent *Pseudomonas chlororaphis* PA23 primes *Brassica napus* defenses through distinct gene networks

**DOI:** 10.1186/s12864-017-3848-6

**Published:** 2017-06-19

**Authors:** Kelly A. Duke, Michael G. Becker, Ian J. Girard, Jenna L. Millar, W. G. Dilantha Fernando, Mark F. Belmonte, Teresa R. de Kievit

**Affiliations:** 1 Department of Microbiology, University of Manitoba, Winnipeg, MB R3T 2N2 Canada; 2 Department of Biological Sciences, University of Manitoba, Winnipeg, MB R3T 2N2 Canada; 3 Department of Plant Science, University of Manitoba, Winnipeg, MB R3T 2N2 Canada

**Keywords:** Biocontrol, *Brassica napus*, Chloroplast, *Pseudomonas chlororaphis* PA23, Reactive oxygen species, RNA-seq, *Sclerotinia sclerotiorum*, Systemic acquired resistance

## Abstract

**Background:**

The biological control agent *Pseudomonas chlororaphis* PA23 is capable of protecting *Brassica napus* (canola) from the necrotrophic fungus *Sclerotinia sclerotiorum* via direct antagonism. While we have elucidated bacterial genes and gene products responsible biocontrol, little is known about how the host plant responds to bacterial priming on the leaf surface, including global changes in gene activity in the presence and absence of *S. sclerotiorum*.

**Results:**

Application of PA23 to the aerial surfaces of canola plants reduced the number of *S. sclerotiorum* lesion-forming petals by 91.1%. RNA sequencing of the host pathogen interface showed that pretreatment with PA23 reduced the number of genes upregulated in response to *S. sclerotiorum* by 16-fold. By itself, PA23 activated unique defense networks indicative of defense priming. Genes encoding MAMP-triggered immunity receptors detecting flagellin and peptidoglycan were downregulated in PA23 only-treated plants, consistent with post-stimulus desensitization. Downstream, we observed reactive oxygen species (ROS) production involving low levels of H_2_O_2_ and overexpression of genes associated with glycerol-3-phosphate (G3P)-mediated systemic acquired resistance (SAR). Leaf chloroplasts exhibited increased thylakoid membrane structures and chlorophyll content, while lipid metabolic processes were upregulated.

**Conclusion:**

In addition to directly antagonizing *S*. *sclerotiorum*, PA23 primes the plant defense response through induction of unique local and systemic defense networks. This study provides novel insight into the effects of biocontrol agents applied to the plant phyllosphere. Understanding these interactions will aid in the development of biocontrol systems as an alternative to chemical pesticides for protection of important crop systems.

**Electronic supplementary material:**

The online version of this article (doi:10.1186/s12864-017-3848-6) contains supplementary material, which is available to authorized users.

## Background

Plants have evolved intricate defense mechanisms to thwart attack from devastating fungal pathogens. Morphological and structural barriers such as a waxy cuticle and tough cell wall are part of an innate defense mechanism against both living organisms and abiotic forces [1, 2]. Successfully bypassing these barriers causes activation of a defense response via detection of microbial- or pathogen-associated molecular patterns (MAMPs/PAMPs), or effector molecules [2, 3]. Activation of MAMP and PAMP pathways are often species-specific and occur through carefully orchestrated signal transduction networks. Following a targeted immune response to PAMPs/MAMPs, systemic resistance may be conferred through two distinct pathways: systemic acquired resistance (SAR) or induced systemic resistance (ISR). While ISR is activated upon colonization of plant roots by nonpathogenic rhizobacteria and fungi, SAR is induced by pathogen attack as well as other elicitors of targeted immune responses [4–6]. The specificity of the response is mediated by changes in gene expression and crosstalk between pathways [7–9]. Nonpathogenic organisms eliciting systemic resistance in plants boost host defense strategies leading to stronger and quicker reactions to future threats [5, 6, 10–13]. Such “priming” of plant defenses involves short- and long-term cellular changes [14].

Plants are exposed to high numbers of nonpathogenic organisms when biocontrol systems are employed. In addition to direct pathogen antibiosis, biocontrol agents (BCAs) can have the added benefit of priming host defenses [12, 15–17]. The majority of bacterial species that exhibit these dual roles are ISR-inducing *Pseudomonas* spp. or *Bacillus* spp. [18]. For example, *Pseudomonas putida* WCS358 suppresses soil-borne pathogens through siderophore-mediated competition for iron, but can also induce ISR in *Arabidopsis thaliana* via host detection of flagellin, pseudobactin and lipopolysaccharides [19]. Such microorganisms are good candidates to replace chemical pesticides, and the number of commercially available BCAs is steadily increasing [18, 20, 21]. In order to successfully implement BCAs in the field, a complete understanding of biocontrol system interactions, including their impact on the host plant, is required.

*Brassica napus* (canola) is an economically important crop of global significance. Despite attempts to breed cultivars with broad resistance traits, canola remains susceptible to a variety of pathogens. The necrotrophic fungus *Sclerotinia sclerotiorum* represents a particularly devastating pathogen to which no immune or highly resistant germplasm has been identified [22, 23]. As the causal agent of canola stem rot, this fungus can infect over 400 plant species worldwide [24]. Such a wide host range has resulted in a heavy reliance on chemical pesticides for managing disease. Because of unwanted effects on the environment along with consumer safety concerns, biological control has emerged as an attractive alternative for crop protection.

*Pseudomonas chlororaphis* PA23 is a BCA capable of preventing *S. sclerotiorum* growth in vitro and *in planta* in susceptible *B. napus* cultivars [25, 26]. We have previously shown that PA23 directly antagonizes *S. sclerotiorum* through the excretion of antifungal metabolites including phenazines, pyrrolnitrin, proteases and lipases [27, 28], with pyrrolnitrin being the primary compound responsible for antagonism [29]. Despite our growing understanding of the molecular mechanisms underlying PA23 antifungal activity, we have yet to understand how the presence of PA23 affects the host organism*.*

In the current study, we examine gene activity genome-wide in leaf tissues of *B. napus* in response to PA23 in the presence and absence of *S. sclerotiorum*. Global RNA sequencing (RNA-seq) was employed to identify differentially expressed genes indicative of *B. napus* defense responses. By itself, PA23 activated gene networks associated with defense priming. Moreover, changes in leaf architecture and increased chlorophyll content were observed in plants treated with PA23 alone. The presence of PA23 diminished *S. sclerotiorum*-induced defense pathways, including production of reactive oxygen species (ROS) and SAR induction. Collectively, these findings show that in addition to direct *S. sclerotiorum* antagonism, PA23 exerts a protective effect through host priming of defense networks.

## Results

### *P. chlororaphis* PA23 reduces *S. sclerotiorum* infection rates in *B. napus*

**Fig. 1:**
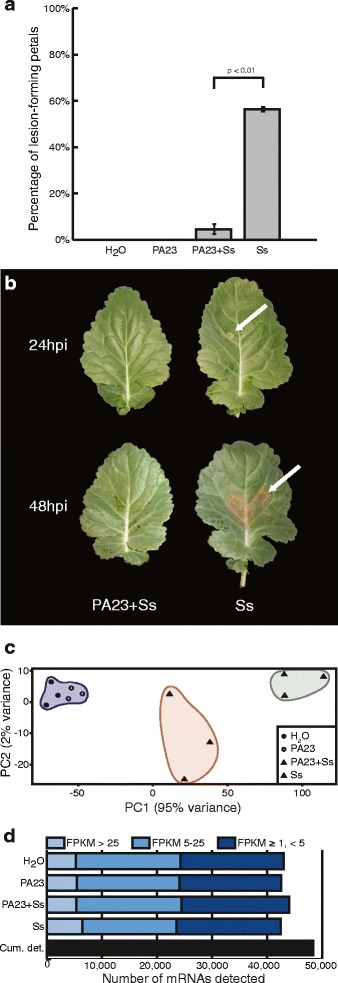
*Brassica napus* infection rates and global transcriptome changes with combinations of PA23 and *Sclerotinia sclerotiorum.* **a** Numbers of lesion-forming petals as a percentage of total petals which fell onto plant leaves in greenhouse assays. **b** *S. sclerotiorum* disease progression on canola leaves at 24 h or 48 h after petal application. PA23 + Ss treatment petals were inoculated with PA23 24 h prior to *S. sclerotiorum* inoculation, whereas Ss treatment petals were inoculated with sterile water. Petals from both treatment groups (PA23 + Ss and Ss) were then infected in vitro with *S. sclerotiorum* 48 h prior to being placed on leaves. **c** Principal component analysis of mRNA sequences from the four treatment groups examined by RNA-seq. Variation between treatments is greater that variation between replicates, and phenotypically similar treatment groups clustered more closely together. **d** Number of unique mRNAs present in treatment groups, as well as the cumulative number of unique mRNA transcripts identified. Transcripts are categorized by frequency of occurrence in the library, as described by the number of fragments per kilobase of transcript per million mapped reads (FPKM) value

To understand how *B. napus* responds to PA23 and how PA23 protects the host plant from *S. sclerotiorum* infection, we compared infection rates at the 30–50% flowering stage in the presence or absence of PA23. When comparing the rate of infection as the proportion of lesion-forming petals to total petals fallen onto the plant canopy, application of PA23 reduced the number of lesions by 91.1% (Fig. 1a) and sustained pathogen suppression for at least 7 days post treatment. In this infection model, leaf necrosis was visible under lesion-forming petals at 24 h post *S. sclerotiorum* infection in plants receiving the pathogen only treatment (Fig 1b).

### Global patterns of gene expression in *B. napus* treated with combinations of PA23 and *S. sclerotiorum*

Next, we studied global patterns of gene activity using RNA-seq to better understand how *B. napus* responds to PA23 in the presence or absence of *S. sclerotiorum*. Principal component analysis (PCA) identified relationships between transcriptomes of plants receiving combinations of PA23 and *S. sclerotiorum*. Biological replicates for each treatment grouped together, with PA23-only treatments grouping closely to the water controls (Fig. 1c). Replicates representing treatment with *S. sclerotiorum* only (Ss) or PA23 + Ss clustered into distinct groups, with Ss clustering farthest from the water control (Fig. 1c).

An average of 81.42% of reads mapped to the *B. napus* cv. Darmor genome (Additional file 1: Table S1). Unmapped reads from all treatments except Ss were composed primarily (58.2%) of *Brassicaceae*, chloroplast, and mitochondrial sequences (Additional file 2: Figure S1). Unmapped reads from the Ss treatment are derived primarily (95.4%) from *Sclerotiniaceae* and other fungi. To identify transcripts that may be unique to *B. napus* cv. Westar we performed a transcriptome assembly of unmapped reads (Additional file 3: Dataset S1). The majority of assembled transcript fragments were identified as *B. oleracea* and suggest cv. Westar may have been recently outcrossed with this species (Additional file 4: Dataset S2).

Figure 1d summarizes mRNA detection and distribution of transcript abundance in treatment groups. A total of 48,454 genes with **F**ragments **P**er **K**ilobase of transcript per **M**illion mapped reads (FPKM) ≥ 1 were detected across all samples, representing 48% of the predicted *B. napus* gene models [30]. The number of *B. napus* transcripts detected was similar across treatments at an average of 43,007 expressed genes. We divided expression levels into low (FPKM ≥1, < 5), moderate (FPKM 5–25), and high (FPKM >25). The proportion of expressed genes falling into each category was similar across all treatment groups (39.0–43.5%, low; 35.3–44.3% moderate; 11.5–13.2%, high), where plants treated with *S. sclerotiorum* alone resulted in the greatest number of highly accumulating transcripts (Fig. 1d). In contrast, the PA23 + Ss treatment group had the greatest number of moderately and lowly accumulating transcripts across treatments.

**Fig. 2:**
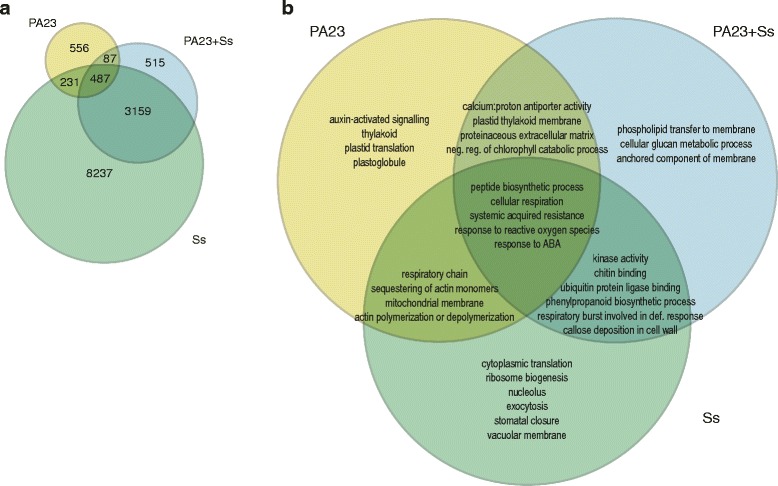
Gene expression changes unique to bacterial or fungal treatments of *Brassica napus* leaves. **a** Venn diagram of *B. napus* gene counts for uniquely and significantly upregulated genes in treatment groups compared to the water control. **b** Venn diagram of enriched GO terms selected from upregulated genes in **a**

Because we observed marked differences on the leaf surface when *S. sclerotiorum* was present with or without PA23, we compared differentially expressed genes (DEGs) of the treatment versus water control to identify similarities and differences at the RNA level. Figure 2a shows shared and specifically-upregulated DEGs in treatment groups compared to the water control. Plants treated with *S. sclerotiorum* alone had the greatest number of specifically-upregulated DEGs at 8237 genes. This trend held for both up- and downregulated genes (Additional file 5: Figure S2A). Plants treated with a combination of PA23 and *S. sclerotiorum* had the fewest specifically-upregulated DEGs at 515 genes, a 16-fold reduction compared to the *S. sclerotiorum* treatment group. The majority of upregulated DEGs observed in PA23 + Ss were shared with the pathogen only (Ss) group (3159 genes). While the number of upregulated DEGs in plants treated with PA23 alone was comparatively small (1361 genes), 556 genes were specific to PA23 treatment alone (Fig. 2a). DEGs of significance from the PA23 treated plants are listed in Table 1. Several markers of systemic acquired response (SAR) including pathogenesis-related proteins *PR-1* (*BnaC03G45470D*) and *PR-2* (*BnaC08G28150D*), lipid transporter protein *DIR1* (*BnaA03G11410D*, *BnaC03G14230D*, *BnaA10G09640D*) and *EARLI1* (*BnaC03g29580D*, *BnaA09g20900D*) are upregulated in the presence of PA23. All DEGs were specific to *B. napus*. When the sequence reads were mapped to the *S. sclerotiorum* genome, *B. napus* tissues treated with *S. sclerotiorum* alone showed an appreciable mapping rate compared to water control, PA23 treatment or PA23 + Ss (Additional file 1: Table S1).

**Table 1:** Genes upregulated in response to PA23 treatment. *B. napus* identifiers used as per the Genome Genoscope Database (www.genoscope.cns.fr/brassicanapus). TAIR identifiers used as per The Arabidopsis Information Resource (TAIR, https://www.arabidopsis.org). Fold change in PA23 compared to the water control

*B. napus* identifier	TAIR identifier	Gene name/function	Fold change
*BnaC03g29580D*	*AT4G12490.1*	EARLI1-like lipid transfer protein 2	53.07
*BnaA09g20900D*	*AT4G12490.1*	EARLI1-like lipid transfer protein 2	40.22
*BnaC07g14090D*	*AT1G22070.1*	TGA3 (Transcriptional activator)	35.95
*BnaC06g37650D*	*AT1G21310.1*	EXT3 (Extensin 3)	32.56
*BnaC06g18380D*	*-*	F-box/LRR-repeat protein 4-like	30.61
*BnaC02g31910D*	*AT5G45890.1*	SAG12 (Senescence-specific cysteine protease)	18.37
*BnaC09g26890D*	*-*	Possible nucleotide phosphorylase	17.43
*BnaC08g46150D*	*-*	Possible malonate decarboxylase	16.69
*BnaC06g14700D*	*-*	Photosystem II reaction center protein A	16.66
*BnaA02g24130D*	*AT5G45890.1*	SAG12 (Senescence-specific cysteine protease)	15.15
*BnaC05g10350D*	*AT1G14080.1*	FUT6 (Fucosyltransferase 6)	14.99
*BnaCnng12890D*	*ATMG00640.1*	ATP4 (ATP synthase subunit 4)	14.18
*BnaA10g18480D*	*AT5G15800.1*	SEP1 (SEPALLATA 1 transcription factor)	12.56
*BnaCnng13130D*	*ATMG00900.1*	CcmC (Cytochrome c assembly protein)	12.04
*BnaC04g28910D*	*AT5G24150.1*	Squalene monooxygenase 1-like	11.94
*BnaAnng01030D*	*AT5G04740.1*	ACR12 (ACT-domain containing protein)	11.88
*BnaCnng24320D*	*AT3G09190.1*	Concanavalin A-like lectin family protein	11.87
*BnaA01g34180D*	*ATCG00130.1*	AtpF (ATP synthase subunit b, chloroplastic)	11.79
*BnaC09g29230D*	*-*	Possible omega-6 fatty acid desaturase	11.24
*BnaAnng35860D*	*AT3G28700.1*	NADH dehydrogenase [ubiquinone] complex I, assembly factor 7-like	9.93
*BnaA01g34980D*	*AT4G19810.1*	ChiC (Class V chitinase)	9.74
*BnaCnng12960D*	*ATMG00070.1*	NAD9 (NADH dehydrogenase subunit 9)	9.64
*BnaA01g33070D*	*AT3G02310.1*	SEP2 (SEPALLATA 2 transcription factor)	9.39
*BnaC05g38940D*	*AT3G14610.1*	Cytochrome P450	8.93
*BnaA03g38630D*	*AT2G14580.1*	Basic PR-1	8.63
*BnaC09g27530D*	*ATCG00340.1*	PsaB (Photosystem I)	8.58
*BnaC04g20930D*	*AT3G30390.2*	Probable amino acid transporter	8.47
*BnaA07g37560D*	*AT5G38100.1*	S-adenosyl-L-methionine-dependent methyltransferases superfamily protein	8.02
*BnaUnng03950D*	*ATMG01170.1*	ATPase subunit 6	7.90
*BnaC04g21290D*	*ATMG01360.1*	COX1 (cytochrome c oxidase subunit 1)	7.87
*BnaC09g32980D*	*AT5G57220.1*	Cytochrome P450, family 81, subfamily F, polypeptide 2	7.77
*BnaA08g22890D*	*AT1G17860.1*	Kunitz family trypsin and protease inhibitor protein	7.73
*BnaA07g21130D*	*-*	Extensin-like protein	7.63
*BnaA01g37280D*	*AT4G11600.1*	GPX6 (Glutathione peroxidase, mitochondrial)	7.49

To identify biological processes activated by the different treatments, we used the custom gene ontology (GO) term enrichment function of ChipEnrich with gene sets identified in Fig. 2a [31]. Figure 2b summarizes GO terms of interest from this analysis. A heatmap of relevant enriched GO terms for genes downregulated in these groups is available in Additional file 5: Figure S2B and the complete list of GO terms with associated *p*-values and genes can be found in Additional file 6: Dataset S3. Response to ROS (log_10_ *p*-value < −4) was significantly enriched for all of the treatments. Subsets of genes belonging to this category accumulated in PA23 + Ss and Ss but not in plants treated with PA23 alone, while others were upregulated in all three treatment groups. In the latter, response to ROS involved upregulation of *FERRETIN 1* (*FER1, BnaC03G00160D*), *FERRETIN 3* (*FER3, BnaC06G15730D*), and two homologs of *HEAT SHOCK TRANSCRIPTION FACTOR A4A* (*HSFA4A, BnaC01G11370D, BnaC03G62890D*). In PA23 + Ss and Ss treatment groups, four homologs of *ASCORBATE PEROXIDASE 1* (*APX1, BnaA06G04380D, BnaA09G49190D, BnaC05G05550D, BnaC08G43490D*), three homologs of *ETHYLENE RESPONSIVE ELEMENT BINDING FACTOR 6* (*ERF6, BnaA01G34910D, BnaA08G08300D, BnaC01G10080D*), two homologs of *XANTHINE DEHYDROGENASE 1* (*XDH1, BnaA09G00610D, BnaCNNG46690D*) and two additional homologs of *HEAT SHOCK TRANSCRIPTION FACTOR A4A* (*HSFA4A, BnaANNG31620D, BnaC07G35520D*) were identified. Similarly, all treatment groups were enriched for the SAR GO term, while the PA23 + Ss and Ss treatment groups were enriched for respiratory burst involved in defense response.

Because reinforcement of the plant cell wall is a marker for priming, it is noteworthy that the PA23 + Ss group was uniquely enriched for GO terms involving cell wall remodeling (phospholipid transfer to membrane, log_10_ *p*-value < −6; anchored component of membrane, log_10_ *p*-value < −5; cellular glucan metabolic process, log_10_ *p*-value < −4) (Fig. 2b). Overrepresented transcripts include lipid transfer proteins and xyloglucan endotransglycosylases. Furthermore, PA23 only and PA23 + Ss groups were both enriched for the proteinaceous extracellular matrix GO term (log_10_ *p*-value < −3).

Several GO terms associated with the chloroplast were overrepresented in PA23-treated plants (Fig. 2b). Specifically, thylakoid (log_10_ *p*-value < −7), plastid translation (log_10_ *p*-value < −4) and plastoglobule (log_10_ *p*-value < −3) were enriched in the PA23 only treatment group, and plastid thylakoid membrane (log_10_ *p*-value < −3) as well as negative regulation of chlorophyll catabolic process (log_10_ *p*-value < −6) were enriched in both PA23 only and PA23 + Ss treatment groups. Transcripts among these groups included subunits of photosystems I and II, plastid-specific ribosomal subunits, and two homologs of the negative regulator of chlorophyll degradation *STAY-GREEN 2* (*SGR2*, *BnaA03G24900D* and *BnaC03G72930D*).

### PA23 prevents the accumulation of ROS in the leaf

**Fig. 3:**
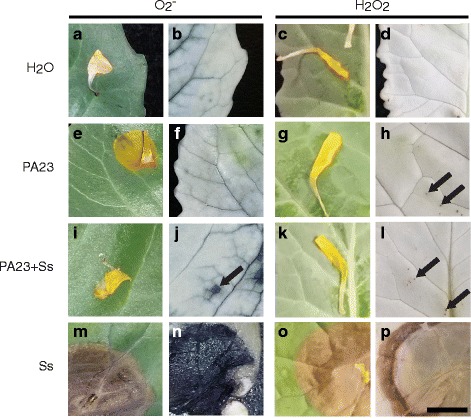
Detection of superoxide radicals (left) and hydrogen peroxide (right) in *Brassica napus* treatment groups. The leftmost column in each set depicts leaves after treatment and before staining. The rightmost column depicts the same area of tissue after petal removal, staining and treatment to remove leaf pigmentation. Scale bar in (P) = 5 mm and is applicable to panels (**a**) - (**p**)

We stained leaf tissues for detection of hydrogen peroxide (H_2_O_2_) and superoxide (O_2_^−^) radicals to help validate the RNA seq dataset. Whereas Ss-treated leaves retained both stains in the regions directly surrounding lesions, indicative of H_2_O_2_ and O_2_^−^ presence (Fig. 3m-p), ROS production was greatly reduced when plants were treated with PA23 prior to the fungal pathogen (Fig. 3i-l). PA23-treated leaves had no regions containing large aggregations of H_2_O_2_ and O_2_^−^, although production of the former appeared similar to the PA23 + Ss treatment group suggesting PA23 activates a mild H_2_O_2_ reaction (Fig. 3g, h).

### Dominant patterns of gene expression reveal treatment-specific responses in *B. napus*

**Fig. 4:**
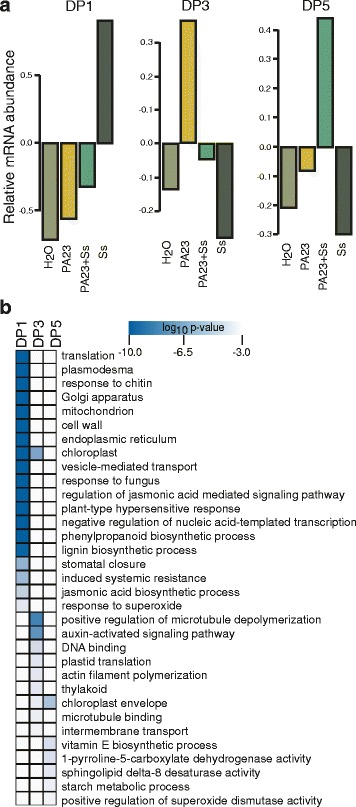
Dominant patterns of gene expression in *Brassica napus* treatment groups. **a** Selection of three dominant patterns of gene expression depicting unique upregulation in one treatment group. **b** Heatmap of enriched GO terms selected from genes identified in the dominant patterns from (**a**)

To identify more complex patterns of expression, we clustered gene activity from all treatment groups into dominant patterns (DPs) of co-expression using a modified fuzzy K means clustering algorithm. We identified six DPs from this analysis (Additional file 7: Figure S3). We focused our attention on three DPs where genes accumulated specifically in response to one of the treatments (Fig. 4a). The number of genes clustering in these DPs ranged from 282 in DP5 to 11,340 in DP1. We identified genes associated with translation, response to fungus, plant-type hypersensitive response, and stomatal closure (log_10_ *p*-value < −8) when plants were infected with *S. sclerotiorum* without protection by PA23 (DP1). The chloroplast GO term is also significantly represented in DP1 (Fig. 4b; log_10_ *p*-value < −28), although no specific chloroplast-related processes were identified. In contrast, pre-treatment with PA23 (DP5) induced chloroplast-related components and processes, such as the chloroplast envelope, vitamin E biosynthesis, and starch metabolic processes (log_10_ *p*-value < −3). When plants were treated with PA23 alone (DP3), genes associated with GO terms for cytoskeletal changes and auxin-activated signaling were upregulated (log_10_ *p*-value < −6), as well as several chloroplast-related terms including plastid translation, thylakoid and chloroplast envelope. Some GO terms were common to more than one DP; in particular chloroplast (DP1 and DP3) and chloroplast envelope (DP3 and DP5), indicating differential expression of genes within these GO terms among treatments (Fig. 4b). A complete list of genes for each DP is available in Additional file 8: Dataset S4.

### PA23 treatment results in structural and metabolic changes in the *B. napus* chloroplast

**Fig. 5:**
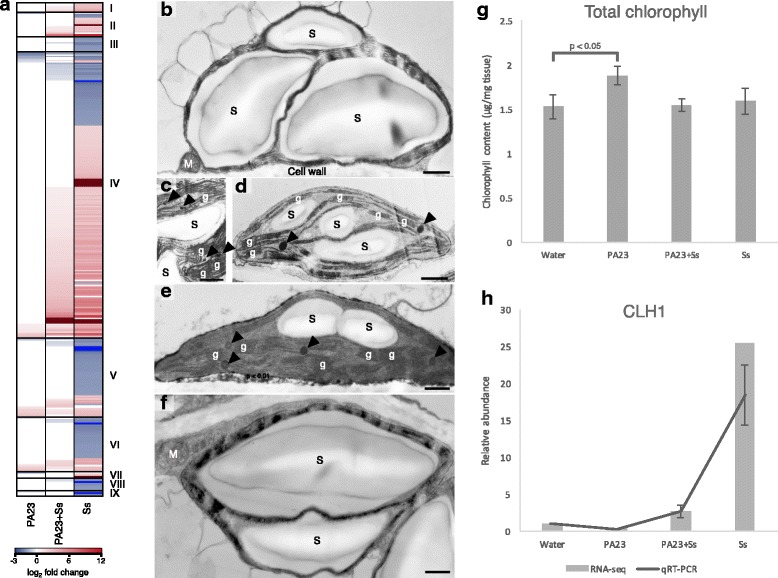
Changes detected in the *Brassica napus* chloroplast in response to combinations of PA23 and *Sclerotinia sclerotiorum*. **a** Differential expression of genes associated with chloroplast-related GO terms, compared to the water control. Only genes with a log_2_ fold change greater than 2 in at least one treatment group are shown. I, chlorophyll catabolite transmembrane transport; II, chloroplast inner membrane; III, chloroplast photosystem I & II; IV, chloroplast stroma; V, chloroplast thylakoid; VI, plastoglobule; VII, protein import into chloroplast stroma; VIII, thylakoid lumen; IX, thylakoid membrane. **b**-**f** Transmission electron micrographs of leaf chloroplasts. **b** Water control, 48 h. **c** PA23, 24 h. **d** PA23, 48 h. **e** PA23 + Ss, 24 h. **f** Ss, 48 h. S = starch granule; g = grana stack; M = mitochondria. Arrows indicate plastoglobules. Scale bar for panels A-E = 500 nm. **g** Chlorophyll b content of treated leaves. **h** Relative abundance of the chlorophyllase 1 transcript as determined by RNA-seq (grey bars) and qRT-PCR (black line)

Since each DP identified a number of chloroplast component- and process-related GO terms, we decided to explore gene expression patterns within this organelle in more detail. The chloroplast GO term was significantly represented in more than one DP indicating differential expression of subsets of associated genes. Accordingly, we compared relative expression levels of genes belonging to chloroplast-related sub-regional and functional GO terms to reveal contrasting expression patterns (Fig.5a). For example, a comparison of relative abundance of genes for chlorophyll catabolite transmembrane transport reveals upregulation in the *S. sclerotiorum*-treated group. Although PA23 + Ss treatment caused many of the same genes to be expressed, transcript abundance was higher in the absence of PA23. It is possible that the reduced infection load associated with the PA23 + Ss group results in decreased transcript abundance. This trend was also observed for the chloroplast inner membrane and chloroplast stroma GO terms (Fig. 5a, II and IV). Genes downregulated in *S. sclerotiorum* -treated plants were enriched for chloroplast photosystem I & II GO terms as well as thylakoid-related GO terms, including genes encoding photosystem I subunits (*PSAN*, *BnaA06g23190D* and *BnaC03g50210D*; *PSAG*, *BnaC06g07480D*; *PSAP*, *BnaC04g51600D*), photosystem II subunits and regulatory proteins (*PSBY*, *BnaA07g38700D* and *BnaC06g26560D*; *PSB27*, *BnaC08g46250D*; *PSBP-1*, *BnaC08g44890D*) and other photosynthesis-related proteins (*CRR23*, *BnaA07g28860D* and *BnaC06g31900D*; *PLASTOCYANIN 2*, *BnaA06g38550D*; *PNSL2*, *BnaA09g45770D*). Overall upregulation of the chloroplast thylakoid and plastoglobule GO terms (Fig. 5a, V and VI) in the PA23 only group was also confirmed. A complete list of gene names and fold change values for genes in Fig. 5a is available in Additional file 9: Dataset S5.

Through transmission electron microscopy (TEM), PA23-mediated changes in chloroplast membrane structure were identified validating our transcriptional findings (Fig. 5b-f). Chloroplasts within the uppermost layer of the palisade mesophyll from the water-treated control group contained large starch granules and reduced thylakoid membrane structure. When plants were treated with PA23, the area dedicated to thylakoid structures increased with a concomitant increase in granal stack organization and the accumulation of plastoglobuli 24 and 48 h post inoculation (Fig. 5c, d). While gene expression in plants treated with PA23 + Ss indicated significant upregulation of starch metabolic processes (Fig. 4b), these chloroplasts were similar in appearance to those of the biocontrol only-treated group containing many grana stacks and plastoglobules (Fig. 5e). When plants were inoculated with *S. sclerotiorum*, the area dedicated to thylakoid structures within the chloroplast appeared to be reduced compared to other treatments (Fig. 5f).

Since structural changes were observed in PA23-treated chloroplasts, we sought to determine whether plant chlorophyll was also affected. Chlorophyll content between treatment groups showed concentrations of total chlorophyll (chlorophyll *a* and *b)* significantly increased by 22.8% when plants were treated with PA23. Conversely, plants exposed to PA23 and *S. sclerotiorum* or *S. sclerotiorum* alone showed no difference in chlorophyll content when compared to the water control (Fig. 5g). To clarify whether these changes were due to increased chlorophyll production or decreased chlorophyll degradation, we examined expression of genes encoding chlorophyllase. Global transcriptional analysis of genes involved in chlorophyll degradation pathways revealed significant downregulation of *CHLOROPHYLLASE 1* (*CLH1*) in PA23-treated plants, which was confirmed via qRT-PCR (Fig. 5h). In contrast, *S. sclerotiorum*-treated plants showed significantly increased levels of *CLH1* and decreased expression of genes associated with multiple chlorophyll-related pathways (Fig. 5h, Additional file 2: Figure S1B).

### PA23 activates unique innate immunity and SAR networks to prime plant defenses

**Fig. 6:**
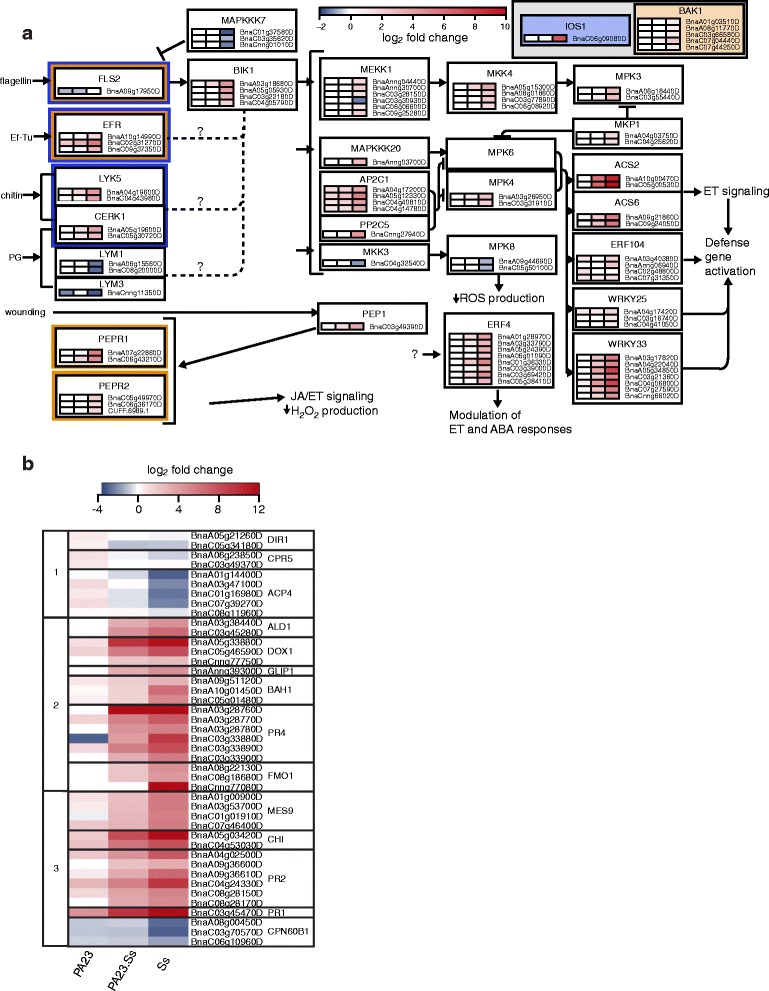
Differentially expressed genes involved in local and systemic defense responses detected within *Brassica napus* treatment groups. **a** Differentially expressed genes involved in innate immunity mapped to known interactions. Expression for each homolog is represented as a heatmap value for each treatment group, from left to right: PA23, PA23 + Ss, Ss. Transcript abundance is measured in log_2_ fold change. Orange and blue borders represent receptors which bind to BAK1 and IOS1 when activated, respectively. **b** Comparison of transcript abundance of select SAR-associated genes as determined by RNA-seq. Transcript abundance is measured in log_2_ fold change. The leftmost column groups genes by expression pattern

To understand how PA23 triggers plant priming mechanisms, we compared the differential accumulation of transcripts known to be involved in innate immunity among treatment groups. The interactions of significantly up- or down-regulated genes encoding products known to function in triggered immunity are presented in Fig. 6a. Treatment-specific expression levels are represented as a heat map for each gene and homolog. Many of these genes were upregulated in response to the pathogen, *S. sclerotiorum*. In response to PA23, two prokaryote-specific surface receptor genes were downregulated, namely *FLS2* (*BnaA09g17950D*), encoding a receptor kinase which binds bacterial flagellin, and *LYM3* (*BnaCnng11350D*), encoding a receptor required for detection of peptidoglycan.

Systemic resistance and defense priming are often the result of activation of these innate immunity networks. As such, we investigated downstream responses to discover how PA23 primes the host plant on a systemic level. The SAR GO term was enriched in all treatment groups, including PA23 alone (Fig. 2b). Analysis of RNA-seq data for genes associated with the SAR GO term revealed genes upregulated in the PA23 only group but downregulated in PA23 + Ss and Ss groups (Fig. 6b subgroup 1). Subgroup 1 contained *DIR1*, *CPR5* and *ACP4,* suggesting that these genes are involved in PA23-induced SAR. We also observed genes induced by *S. sclerotiorum* only (Fig. 6b subgroup 2), as well as those showing altered expression in all treatment groups compared to the water control (Fig. 6b, subgroup 3). Treatment with *S. sclerotiorum* alone induced the expression of *ALD1*, *GLIP1*, *PR4* and *DOX1*. The genes *MES9*, *PR1*, *PR2* and *CHI* were upregulated in all treatment groups, although expression was higher in plants where *S. sclerotiorum* was present. The same trend could be observed for the downregulation of *CPN60B1*. Expression levels for several of the genes mentioned above (*ALD1*, *CHI*, *DOX1*, *FMO1*, *PR1*, and *PR4*) were confirmed using qRT-PCR (Additional file 10: Fig. S4).

## Discussion

Many nonpathogenic rhizobacteria are capable of priming plants for an enhanced defense response via induced systemic resistance (ISR) or systemic acquired resistance (SAR) (see 5,6 and [32] for reviews). However, few of these nonpathogenic microorganisms have been shown to directly antagonize pathogens, especially when applied to the plant phyllosphere [17]. In the current study, we provide compelling evidence that in addition to direct antagonism of *S. sclerotiorum* mitigating disease progression, PA23 protects *B. napus* through priming of host defense systems at the RNA level.

The *B. napus*-*S. sclerotiorum* pathosystem has recently been transcriptionally profiled under a variety of conditions, providing new insight into genetic processes behind hormone and defense pathway signalling changes that support plant innate resistance [33, 34]. Patterns of gene expression revealed that SAR is weakly activated in plants when treated with the biocontrol agent PA23. The activation of an immune response in PA23-treated plants was reflected in both clustering analysis and in the number of DEGs among groups (Figs. 1c, 2a, Additional file 2: Figure S1A). Given the majority of DEGs in the PA23 + Ss group were shared with the Ss group, we hypothesize that *S. sclerotiorum* triggers many of the same defense mechanisms in *B. napus* regardless of PA23. It is important to note, however, that the magnitude of the response is lessened by the presence of the bacteria. This reduction likely results from PA23 antibiosis controlling the *S. sclerotiorum* infection. Additionally, PA23 may attenuate the host defense response through detection of bacterial effectors leading to priming.

Plant cellular signaling cascades which activate defense priming mechanisms are modulated by PAMP/MAMP-detecting pattern recognition receptors (PRRs). Wu al. (2016) observed differential activation of PRRs between resistant and susceptible lines of canola, highlighting the role of PRR activation in response to *S. sclerotiorum*. In plants treated with PA23 alone, surface detection of this nonpathogenic bacterium culminates in systemic defense stimulation via SAR. Genes associated with PAMP/MAMP- and DAMP-triggered immunity networks were turned on in response to *S. sclerotiorum*, but not PA23 (Fig. 6a). The downregulation of *FLS2* in plants treated with PA23 alone is consistent with studies showing that FLS2 is degraded after initial ligand binding to prevent continuous stimulation [35]. Thus, it is logical to surmise that detection of PA23 flagellin by the FLS2 receptor complex contributes to downstream induced defense processes. A similar mechanism may be responsible for repression of *LYM3*. Willmann et al. [36] found that in Arabidopsis, infection with virulent *P. syringae* pv*. tomato* DC3000 lead to strong downregulation of *LYM3, FLS2* and to a lesser degree *LYM1*. *LYM1* activity is unchanged in response to PA23. Differences in *LYM1* and *LYM3* expression may indicate receptor specificity, as these two proteins are not functionally redundant [36]. The role these receptors play in the activation of systemic resistance appears to be variable. For example, flagellin plays an important role in the induction of systemic defense in response to enteric bacteria applied to leaves of Arabidopsis [37]. In the rhizosphere, purified flagellin from nonpathogenic *P. putida* WCS358 induced ISR in Arabidopsis but not in bean or tomato [19]. Moreover, *Pseudomonas fluorescens* SS101 induced systemic resistance in Arabidopsis *fls2* mutants suggesting that FLS2 is not required for priming during this interaction [38]. In the future, determining the roles of these receptor complexes in PA23-mediated defense priming should provide additional insight into the molecular underpinnings of this tri-partite system at the plant cell surface.

SAR is a classic example of defense priming which is usually associated with localized pathogen attack [14]. While genes associated with SAR were induced in all treatments, we identified genes specifically upregulated in plants treated with PA23 alone. Elevated *DIR1*, *CPR5*, and *ACP4* activity are indicative of SAR induction via glycerol-3-phosphate (G3P). G3P is one of several inducers of SAR, and is also a precursor for the synthesis of membrane and storage lipids [39, 40]. The lipid transfer protein, DIR1, which is upregulated in PA23-treated plants, leads to G3P accumulation and G3P-induced SAR [41]. DIR1 binds to EARLI1, a paralog of AZI1 which is required for SAR and upregulated in PA23-only treated plants (Table 1) [42]. Expression patterns of *ACP4* further support this hypothesis, as ACP4 knockout plants are unable to perceive mobile SAR signals [43]. ACP4 also plays a major role in the biosynthesis of fatty acids within the chloroplast, which indirectly increases G3P levels [41, 44]. While CPR5 is thought to have a role in inducing SAR, its exact function has not been defined. Notwithstanding, CPR5 has been shown to negatively regulate programmed cell death caused by effector-triggered immunity, which supports the hypothesis that PA23 promotes plant growth, as it is known that *S. sclerotiorum* infection results in cell death [45–47]. Taken together, our data support a role for G3P in PA23-mediated defense priming which serves to protect plants from fungal infection.

As a downstream marker of immune responses, ROS function as part of a localized hypersensitive-type reaction to invading pathogens, as well as signals for systemic response initiation [48]. Sclerotinia acutely modifies the redox state of the host at infection sites to promote pathogenesis through production of oxalic acid [49]. Several ROS response genes were identified that are upregulated in either all treatment groups (PA23, PA23 + Ss, Ss), or in those exclusively treated with *S. sclerotiorum* (Ss, PA23 + Ss), similar to the activation of redox homeostasis genes observed by Yang et al. [50]. *HSFA4A* is thought to both regulate plant responses to oxidative stress and function as an antiapoptotic factor [51]. Upregulation in PA23 groups suggests that treatment with bacteria alone can induce the plant oxidative stress response resulting in ROS production. This reaction appears to be mild in nature, as only two of the four upregulated HSFA4A homologs accumulated in the PA23 treatment group. In addition, HSFA4A-induced *ASCORBATE PEROXIDASE 1* (*APX1*) was upregulated in *S. sclerotiorum*-treated plants, and is required for H_2_O_2_ scavenging and the prevention of protein oxidation during oxidative stress [52]. Regulation of plant defense through ROS in the presence of PA23 may operate via the expression of *ETHYLENE RESPONSE FACTOR 6* (*ERF6*) and *XANTHINE DEHYDROGENASE 1* (*XDH1*). ERF6 modulates the expression of antioxidant enzymes to control ROS production and XDH1 is thought to be a source of O_2_^−^ production [48, 53]. An important difference between the PA23 + Ss and Ss groups is upregulation of superoxide dismutase activity in DP5, which suggests enhanced management of O_2_^−^ molecules in PA23 + Ss (Fig. 4a, b). Thus, gene expression changes indicate a mild and controlled production of ROS in response to PA23 alone, while pretreatment with PA23 prior to pathogen exposure allows for enhanced management of the oxidative stress response.

To substantiate the changes observed at the RNA level, we were interested to see if PA23 stimulates ROS production and/or modulates ROS levels on the leaf surface. In plants treated with PA23 alone, ROS production involving low levels of H_2_O_2_ was observed (Fig. 3). This is significant as non-toxic levels of H_2_O_2_ are key to the activation of priming pathways which reinforce resistance to abiotic and biotic stressors [54]. Large regions staining for both H_2_O_2_ and O_2_^−^ molecules in Ss-treated leaves indicates a widespread oxidative stress response which appears to be unregulated. Leaves treated with PA23 + Ss had not only H_2_O_2_ but O_2_^−^ as well, confirming that *S. sclerotiorum* induces an oxidative stress response involving both molecules. Conversely, O_2_^−^ staining was not observed in the PA23 only treatment group (Fig. 3f, j). Collectively, our findings establish that ROS production in PA23-only treated leaves is limited to mild oxidative stress and is likely an outcome of weakly induced upstream innate immunity. Such findings support a role for ROS in the priming of plant basal defenses and initiation of long-distance signaling consistent with downstream PA23-mediated induction of SAR genes. In response to *S. sclerotiorum,* ROS production is limited in plants pretreated with PA23. We believe this results from a combination of direct fungal antagonism by PA23 and increased capacity to maintain ROS homeostasis during oxidative stress.

Thylakoid membranes in chloroplasts are a major source of ROS because they house the photosynthetic electron transport system [55]. Oxidative stress observed during *S. sclerotiorum* infection is likely responsible for damage to the chloroplast and a reduction in photosynthetic activity [56, 57]. In Ss-treated plants, the observed upregulation of *CLH1* is likely due to chloroplast damage leading to the release of chlorophylls from thylakoid membranes. *CLH1* expression is induced for quick degradation of these photoactive molecules, a process known to be elicited by necrotrophic pathogens [58, 59]. TEM imaging substantiated these findings, as the region containing thylakoid tissue was decreased. In contrast, we observed an increase in relative area dedicated to thylakoid structures in chloroplasts within PA23-treated plants coupled with an increase in total chlorophyll content and repression of *CLH1.* In other studies, BCA application has been associated with increased chlorophyll in the plant host [60–62]. The lack of overexpression of *CLH1* in the PA23-only treatment group is consistent with evidence showing that *CLH1* is only induced after cellular collapse, a defense response not elicited by nonpathogenic organisms [63]. Moreover, active repression of *CLH1* may be evidence of a yet-to-be-identified role for chlorophyll in defense priming. Detection of more plastoglobules in the chloroplasts of PA23-treated plants suggests increased plastid lipid metabolism, potentially facilitating synthesis of lipid signaling molecules and remodeling of thylakoid membranes [64].

Such gene expression patterns together with the observed morphological changes in the chloroplasts of PA23-treated leaves correlate to the activation of SAR-modulated expression and mitigation of *S. sclerotiorum* disease progression.

Finally, the plant cell wall is an important component of innate immunity and defense priming [65]. Changes in gene expression related to cell wall architecture were exclusively observed for plants in the PA23 + Ss treatment group. GO terms associated with cell wall development including thirteen xyloglucan endotransglycosylases which function to build up the cell wall during cell growth [66] and three homologs of *LIPID TRANSFER PROTEIN 2* (*LTP2*) were enriched. These proteins may be involved in the deposition of cutin or wax in the extracellular matrix, and may increase tolerance to pathogens as has been shown in tobacco [67]. As these genes were only upregulated in PA23 + Ss, we hypothesize this to be the result of a heightened defense response to *S. sclerotiorum* primed by PA23 pretreatment.

## Conclusions

Findings from the current study broaden our understanding of PA23-mediated control of sclerotina stem rot. Beyond antibiosis, application of PA23 to the plant phyllosphere protects canola through induction of plant innate-immune pathways involving G3P-mediated SAR, ROS signaling molecules and protection of chloroplast integrity. Together, these processes serve to prime the plant and enhance defenses in response to fungal attack. As we move towards more sustainable approaches for crop disease management, it is essential that we fully appreciate the impact that BCAs pose on the plants they protect as well as the surrounding environment. Findings from the current study are an important step in this direction.

## Methods

### Plant and bacterial growth conditions

*Brassica napus* cv. Westar plants were grown in Sunshine Mix #1 soil in growth chambers at 21 °C with a light/dark photoperiod of 16 h/8 h and 0% humidity. *P. chlororaphis* PA23 was grown overnight in Luria-Bertani broth at 28 °C in a shaking incubator.

### Greenhouse infection assays

One day prior to fungal pathogen exposure, *B. napus* plants at the 30% flowering stage were sprayed until dripping with a 2 × 10^8^ cfu/mL solution of PA23 resuspended in sterile water supplemented with 0.02% Tween 20 as a surfactant. Plants not receiving biocontrol treatment (water control and Ss only groups) were sprayed with sterile water (0.02% Tween 20). Plants were sealed in clear bags to maintain relative humidity and returned to the growth chamber for 24 h. The following day, bags were removed and plants receiving the pathogen treatment were sprayed with an 8 × 10^4^ spores/mL solution of *S. sclerotiorum* ascospores resuspended in sterile water (0.02% Tween 20). Control plants and plants to be exposed only to PA23 were sprayed with sterile water (0.02% Tween 20). Plants were transferred to a humidity chamber with humidity levels of 70–90% for 72 h. During this time, plants were gently shaken at the base twice to encourage petals to detach and fall into the plant canopy. Infection rates were quantified by calculating the ratio of petals causing lesions to total petals in the plant canopy. Counts from three plants were pooled for each treatment. This experiment was performed three times.

### RNA extraction and sequencing

Infection assays were carried out as described above, and tissue from three biological replicates was collected for RNA extraction. Three leaves per plant and three plants per treatment group were used for each biological replicate. Leaves upon which petals had landed were used for collection, as these are sites of potential infection. The petal was removed from the leaf and approximately 1cm^2^ area of leaf tissue surrounding the site was collected with a scalpel. For *S. sclerotiorum*-infected leaves, green tissue immediately surrounding the lesion was collected. Cuttings were flash frozen in liquid nitrogen, and stored at −80 °C for no more than 2 days before processing. Total RNA was extracted using PureLink® Plant RNA Reagent (Invitrogen). DNA contamination was removed with the Turbo DNA-*free*™ kit (Ambion), following the manufacturer’s instructions. RNA concentration was verified using a NanoVue spectrophotometer (GE Healthcare), and quality was measured with an Agilent 2100 Bioanalyzer with Agilent RNA 6000 Pico and Nano Chips (Agilent Technologies; Santa Clara, CA, USA). RNA-seq libraries were prepared according to the alternative HTR protocol (C2) described by Kumar [68] with the exception of PCR enrichment of the libraries, where the number of cycles was adjusted to 11. Libraries were validated using the Agilent Bioanalyzer High Sensitivity DNA Assay with DNA chips (Agilent Technologies). The desired fragment sizes of sheared cDNA with ligated adapters were isolated employing the E-Gel® electrophoresis system (Invitrogen). 100 bp single-end RNA sequencing was carried out at Génome Québec (Montreal, Canada) on the Illumina HiSeq 2000 platform.

### Data analysis

Sequenced reads were analyzed to remove barcode adapters and low quality reads using the Trimmomatic tool [69]. The parameters for Trimmomatic which maximized mapping efficiency to the *B. napus* and *S. sclerotiorum* genomes (*B. napus*: v.4.1, Chalhoub et al. [30]; *S. sclerotiorum*: v1, Amselem et al., [70]) were determined using FastQC reports for quality control (http://www.bioinformatics.babraham.ac.uk/projects/fastqc/) followed by alignment with Tophat2 v.2.1.0 [71]. Reads mapped to the *B. napus* and *S. sclerotiorum* genomes as expected, with 81.42% of reads mapping to *B. napus* across samples (Additional file 1: Table S1). Multiple mapping reads were retained to detect expression of plant defense and resistance genes of interest that may be duplicated within the genome. The value of multiple-mapping reads is determined by dividing the count by the total number of hit locations [71]. For example, a read mapping to four locations will be recorded as 0.25 at each position. This correction statistically favors uniquely mapping reads in downstream analyses. Alignment of reads to these genomes was performed in high-sensitivity mode using *B. napus* reference annotation v5 from Chalhoub et al. [30] and the *S. sclerotiorum* reference annotation from Amselem et al. [70] as guides. The cufflinks and cuffmerge tools within the Cufflinks v.2.2.1 suite [71] were employed to construct a transcriptome from the reads and identify novel transcripts. Transdecoder (https://transdecoder.github.io) was used to identify open reading frames (ORFs) within transcript sequences. Genes were identified by aligning translated ORF sequences with proteins in the Arabidopsis TAIR10, NCBI and Uniprot databases using BLAST [72]. Read counts were normalized to FPKM values using the cuffquant and cuffdiff tools in the Cufflinks package with default settings [71]. Significantly differentially expressed genes were identified as those with a corrected *p*-value <0.05 (false discovery rate = 0.05). This output was used for hierarchical clustering via the pvclust package (https://cran.r-project.org/web/packages/pvclust/pvclust.pdf) and Venn diagram generation via Venny v2.1 (http://bioinfogp.cnb.csic.es/tools/venny/index.html). Dominant patterns (DPs) of expression were identified using the cuffdiff output data via the Fuzzy K-means (FKM) implementation FANNY (https://cran.r-project.org/web/packages/cluster/cluster.pdf) with a K value of 10. Transcripts with a Pearson’s correlation of 0.85 or above were assigned to DPs. Principal component analysis was performed on raw counts using the DESeq2 package [73]. Unmapped reads were converted from BAM format to FASTA with the SAMtools software package (http://samtools.sourceforge.net/). Fasta files were then aligned to the SILVA phylogenetic database (https://www.arb-silva.de/) with a local instance of ncbi-BLAST-2.6.0+ (https://blast.ncbi.nlm.nih.gov/Blast.cgi) to identify the composition of unmapped reads (Additional file 2: Figure S1). To identify genes that may be unique to cv. Westar, unmapped reads from water and PA23 control samples were assembled to transcript fragments (Additional file 3: Dataset S1) with the Trinity package (https://github.com/trinityrnaseq/trinityrnaseq/wiki). Assembled transcripts were then aligned with a local instance of ncbi-BLAST-2.6.0+ to a database of *Brassicaceae* transcript sequences (Additional file 4: Dataset S2).

### Staining for reactive oxygen species

*B. napus* leaves were stained for hydrogen peroxide (H_2_O_2_) and superoxide radical (O_2_^−^) accumulation as per the methods of Kumar et al. [68]. Briefly, leaves were severed with petioles intact and immersed in staining solution overnight in the dark, while avoiding contact between the solution and the severed petiole to reduce staining of the vein conduits. For H_2_O_2_ detection, a 1 mg/mL solution of 3,3′-diaminobenzidine was used. A 0.2% solution of nitrotetrazolium blue chloride in 50 mM sodium phosphate buffer at pH 7.5 enabled identification of O_2_^−^. The following day, chlorophyll was removed from the leaves by submersion in 95% ethanol and heating in a boiling water bath.

### Chlorophyll quantification

Concentrations of chlorophyll *a* and *b* were determined based on methods described by Arnon [74] and Porra [75]. Briefly, tissue from two leaves per plant was combined as one biological replicate and ground in liquid nitrogen. Approximately 200 mg of powder was extracted in 80% acetone buffered to pH 7.8. All steps of the extraction were performed in the dark. Concentrations were calculated as described by Porra [75]. Three biological replicates were collected and absorbance values were measured twice for each replicate. The experiment was repeated twice.

### Transmission electron microscopy

Leaf tissue was collected as above and processed following the methods of Chan and Belmonte [76]. Tissue was fixed overnight in 3% glutaraldehyde in 0.025 M cacodylate buffer supplemented with 5 mM calcium chloride (pH 7.0). Plant material was rinsed with cacodylate buffer and post-fixed with 2% osmium tetroxide in 0.8% KFe(CN)_6_ in cacodylate buffer. After post-fixation, the sample was rinsed with distilled water and stained overnight with a 0.5% aqueous uranyl acetate solution. Plant material was rinsed in distilled water and dehydrated in a graded ethanol series. The leaf tissue was further dehydrated in 1:1 absolute ethanol to propylene oxide (*v:v*) followed by 100% propylene oxide. Finally, tissue was gradually infiltrated and embedded in Spurr’s epoxy resin at 70 °C. All methods prior to embedding were performed at 4 °C. Using a Reichert–Jung Ultracut ultramicrotome, sections were cut (90 nm thickness) with a Diatome diamond knife and mounted on copper grids. The sections were visualized with a Hitachi H-7000 transmission electron microscope at 75 kV and pictures were taken using AMT Image Capture Engine version 601.384.

### Differential gene expression verification using qRT-PCR

RNA collected for RNA sequencing was also used to synthesize cDNA for qRT-PCR. qRT-PCR primers are listed in Additional file 11: Table S2. Following integrity checks via the Agilent 2100 Bioanalyzer with Agilent RNA 6000 Pico and Nano Chips (Agilent Technologies; Santa Clara, CA, USA), cDNA was synthesized with the Maxima First Strand cDNA Synthesis Kit (Thermo Fisher Scientific, Inc.). qRT-PCR was performed on a Bio-Rad CFX Connect™ Real-Time System with SsoFast™ EvaGreen® Supermix (Bio-Rad, USA). The following cycling conditions were employed: 95 °C for 30 s, followed by 45 cycles of 95 °C for 2 s and 60 °C for 5 s. Melt curves (0.5 °C increments in a 68–90 °C range) for each gene were performed to assess the sample for non-specific targets, splice variants and primer dimers. CFX Manager v3.1 (Bio-Rad, USA) was used to calculate relative mRNA abundance from three technical replicates per primer set per sample utilizing the ΔΔCt method. ATGP4 was incorporated as an endogenous control and water-treated tissue was used as a reference sample. Three biological replicates for each treatment were measured.

## Additional files

Additional file 1: Table S1. RNA-seq library reads mapped to the *Brassica napus* and *Sclerotinia sclerotiorum* genomes. (PDF 14.7 kb)

Additional file 2: Figure S1. Identification of contamination within unmapped reads with ncbi-BLAST. For all treatments, unmapped reads were converted to fasta format with SAMtools and aligned to the SILVA rRNA sequence database. Average distribution of alignments are represented in individual pie charts for each treatment group. (PDF 10 kb)

Additional file 3: Dataset S1. Trinity transcriptome assembly of unmapped reads from control treatment groups. (FASTA 51567 kb)

Additional file 4: Dataset S2. ncbi-BLAST results of unmapped transcripts assembled with Trinity to Brassicaceae nucleotide database. (XLSX 3895 kb)

Additional file 5: Figure S2. Downregulated genes in canola treatment groups. A. Venn diagram of *B. napus* gene counts for uniquely and significantly downregulated genes in treatment groups compared to the water control. B. Heatmap of enriched GO terms selected from genes identified in A. (PDF 347 kb).

Additional file 6: Dataset S3. GO terms enriched in shared and uniquely-expressed genes between treatment groups, with associated log_10_ *p*-values for each treatment. List of GO terms was generated via analysis of lists of genes grouped from Fig. 2a in CanEnrich software. Red-highlighted cells indicate significant *p*-values of less than 0.001. (XLSX 968 kb)

Additional file 7: Figure S3. Dominant patterns of expression generated from RNA-seq FPKM values using a Fuzzy K-means clustering algorithm. (PDF 293 kb)

Additional file 8: Dataset S4. List of genes comprising each dominant pattern (DP) of expression seen in Additional file 2: Figure S2. (XLSX 291 kb)

Additional file 9: Dataset S5. Gene names, log_2_ fold change values and corresponding gene ontology (GO) term for select chloroplast-related GO terms. (XLSX 29 kb)

Additional file 10: Figure S4. Relative abundance of select SAR-related gene transcripts as determined by RNA-seq (grey bars) and qRT-PCR (black line). (PDF 100 kb)

Additional file 11: Table S2. qRT-PCR primers used in this study. (PDF 14.7 kb)

## Abbreviations

BCA: Biocontrol agents
Canola: *Brassica napus*
DEGs: Differentially expressed genes
DP: Dominant pattern; transmission electron microscopy (TEM)
FKM: Fuzzy K-means
FPKM: Fragments Per Kilobase of transcript per Million mapped reads
G3P: Glycerol-3-phosphate
GO: Gene ontology
H_2_O_2_: Hydrogen peroxide
ISR: Induced systemic resistance
MAMPs: Microbial-associated molecular patterns
O_2_^−^: superoxide
PAMPs: Pathogen-associated molecular patterns
PCA: Principal component analysis
PGPR: Plant growth-promoting rhizobacteria
PRRs: PAMP/MAMP-detecting pattern recognition receptors
RNA-seq: RNA sequencing
ROS: Reactive oxygen species
SAR: Systemic acquired resistance
Ss: *S. sclerotiorum*
TAIR: The Arabidopsis Information Resource
