# Supplementary material for: The biocontrol agent Pseudomonas chlororaphis PA23 primes Brassica napus defenses through distinct gene networks
Source: BMC Genomics. 2017 Jun 19;18:467. doi: 10.1186/s12864-017-3848-6 (PMC5477169; doi:10.1186/s12864-017-3848-6)
Supplement: Supplementary file 1 — RNA-seq library reads mapped to the Brassica napus and Sclerotinia sclerotiorum genomes. (PDF 14.7 kb) [file 12864_2017_3848_MOESM1_ESM.pdf]

Table S1. RNA-seq library reads mapped to the *Brassica napus* and *Sclerotinia sclerotiorum* genomes.

Treatment (Replicate)	Total reads	Reads mapped to *B. napus* genome	*B. napus* mapping %	Reads mapped to *S. sclerotiorum* genome	*S. sclerotiorum* mapping %
H_2O (1)	53,994,750	39,109,273	78.14%	46,038	0.09%
H_2O (2)	46,151,214	33,249,878	77.65%	33,761	0.08%
H_2O (3)	33,188,701	26,792,764	83.31%	24,608	0.08%
PA23 (1)	13,165,652	10,600,591	84.09%	14,545	0.12%
PA23(2)	28,819,289	23,359,660	83.80%	21,184	0.08%
PA23 (3)	23,310,938	18,915,639	83.78%	17,072	0.08%
PA23+Ss (1)	42,652,467	30,549,663	77.39%	34,130	0.09%
PA23+Ss (2)	29,392,010	23,728,001	83.48%	53,276	0.19%
PA23+Ss (3)	37,445,373	28,861,605	82.50%	358,553	1.02%
Ss (1)	28,442,683	22,522,734	82.03%	654,286	2.38%
Ss (2)	46,092,745	32,405,839	80.93%	1,143,800	2.86%
Ss (3)	23,707,533	18,188,028	79.97%	676,368	2.97%
