# Supplementary figures and images for: The biocontrol agent Pseudomonas chlororaphis PA23 primes Brassica napus defenses through distinct gene networks
Source: BMC Genomics. 2017 Jun 19;18:467. doi: 10.1186/s12864-017-3848-6 (PMC5477169; doi:10.1186/s12864-017-3848-6)

H2O

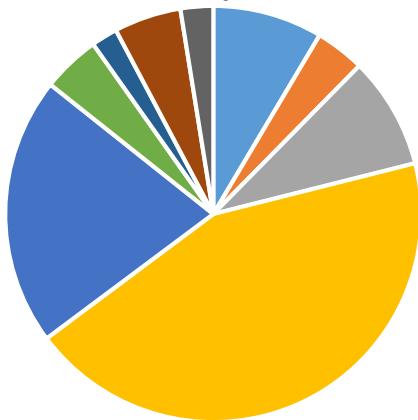

SS lesion

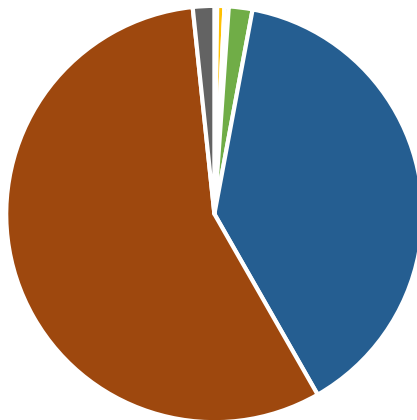

PA23

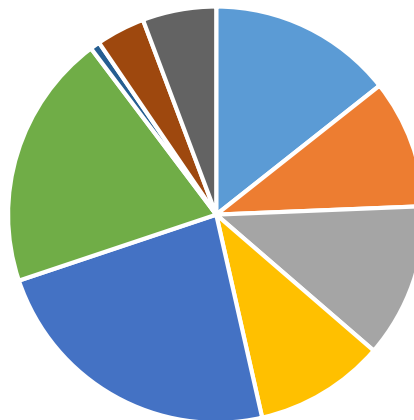

PA23/SS

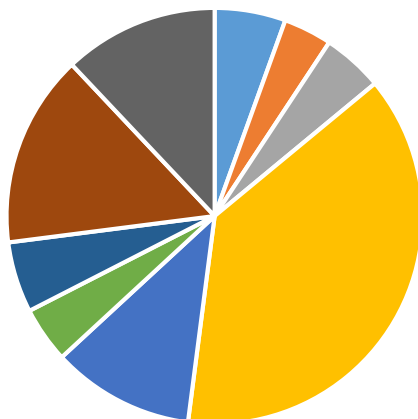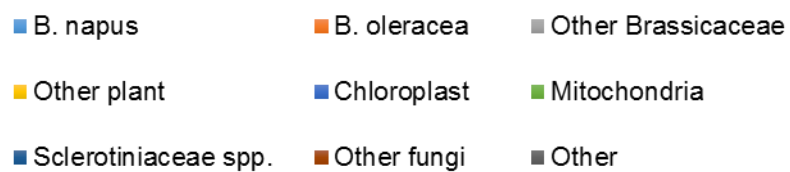

Supplement: Supplementary file 2 — Identification of contamination within unmapped reads with ncbi-BLAST. For all treatments, unmapped reads were converted to fasta format with SAMtools and aligned to the SILVA rRNA sequence database. Average distribution of alignments are represented in individual pie charts for each treatment group. (PDF 10 kb) [file 12864_2017_3848_MOESM2_ESM.pdf]

**A**

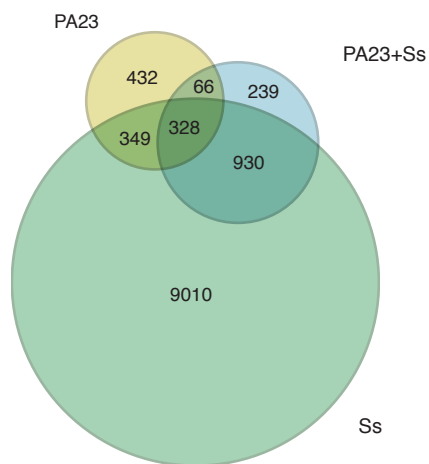

**B**

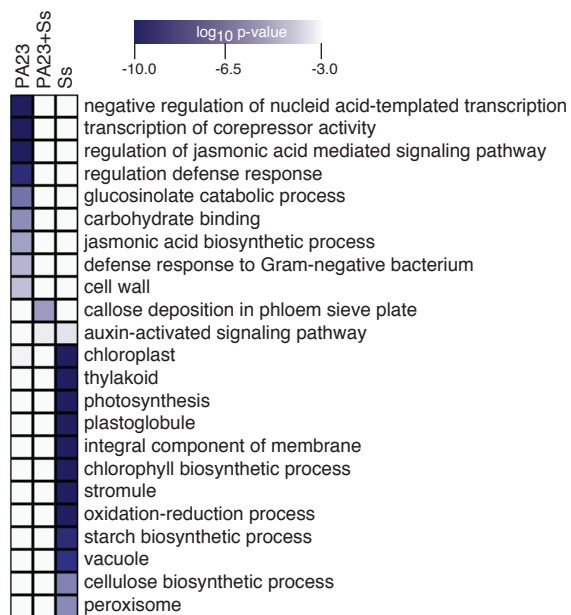

Supplement: Supplementary file 5 — Downregulated genes in canola treatment groups. A. Venn diagram of B. napus gene counts for uniquely and significantly downregulated genes in treatment groups compared to the water control. B. Heatmap of enriched GO terms selected from genes identified in A. (PDF 347 kb). [file 12864_2017_3848_MOESM5_ESM.pdf]

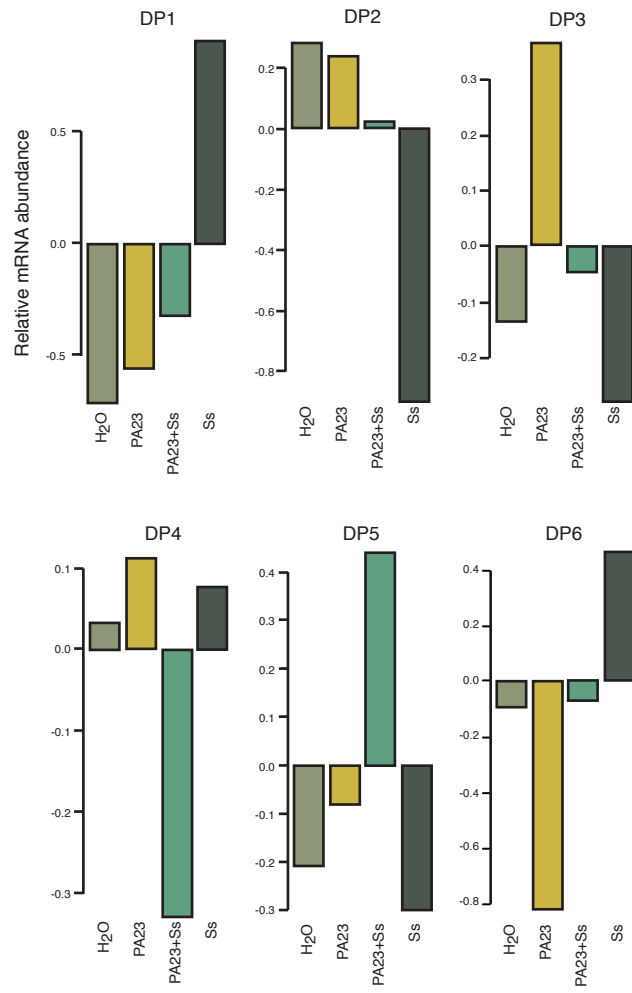

Supplement: Supplementary file 7 — Dominant patterns of expression generated from RNA-seq FPKM values using a Fuzzy K-means clustering algorithm. (PDF 293 kb) [file 12864_2017_3848_MOESM7_ESM.pdf]

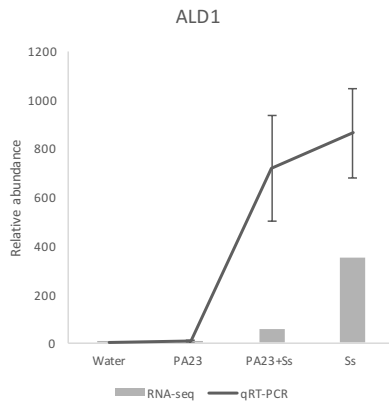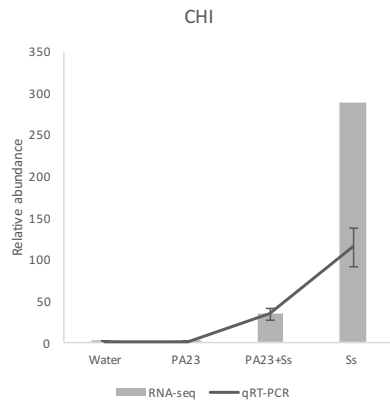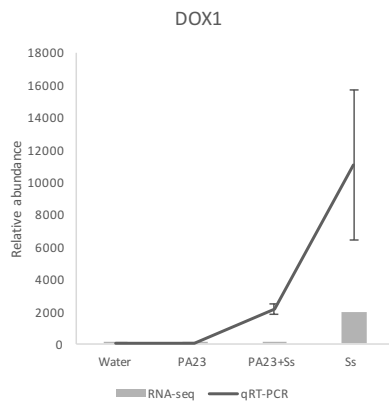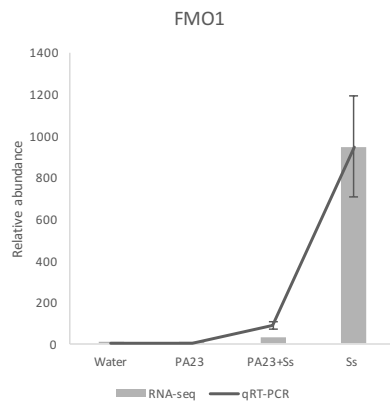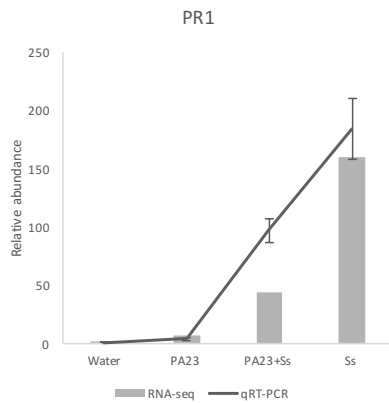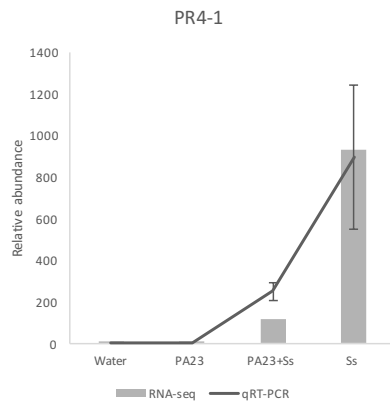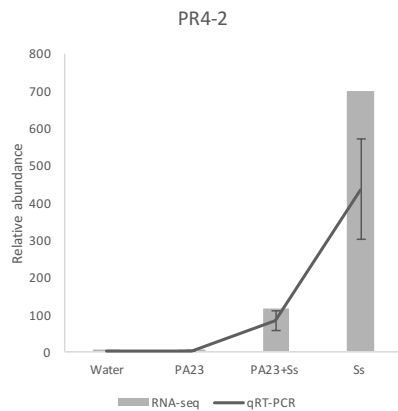

Supplement: Supplementary file 10 — Relative abundance of select SAR-related gene transcripts as determined by RNA-seq (grey bars) and qRT-PCR (black line). (PDF 100 kb) [file 12864_2017_3848_MOESM10_ESM.pdf]
