# Supplementary material for: The biocontrol agent Pseudomonas chlororaphis PA23 primes Brassica napus defenses through distinct gene networks
Source: BMC Genomics. 2017 Jun 19;18:467. doi: 10.1186/s12864-017-3848-6 (PMC5477169; doi:10.1186/s12864-017-3848-6)
Supplement: Supplementary file 11 — qRT-PCR primers used in this study. (PDF 14.7 kb) [file 12864_2017_3848_MOESM11_ESM.pdf]

Table S2. qRT-PCR primers used in this study.

Primer name	Oligonucleotide sequence	Gene name
ALD1both-F	5′- CCGAAGCAGATCACCTCAGA -3′	ALD1
ALD1both-R	5′- CTTGTCACCTTGTTCCAGGC -3′	ALD1
BnaA05g03420D-F	5′- GCTCAAGACCAGGTTCTTGC -3′	CHI
BnaA05g03420D-R	5′- CCTTGATTGTCGGGCCAAAG -3′	CHI
BnaA06g13830D-F	5′- GCGAGGCTTGATCCTTTGC -3′	CLH1
BnaA06g13830D-R	5′- AGCTTCCCTGAGGATACCAA -3′	CLH1
BnaA05g33880D-F	5′- ACGAGTGTCCCTTAAGCTCC -3′	DOX1
BnaA05g33880D-R	5′- GGGTGTACGGGAATTAAGCG -3′	DOX1
FMO1-F	5′- AAGAAAGTCGCGGTCATTGG -3′	FMO1
FMO1-R	5′- TCCACCTTCTCCTTGATTTGC -3′	FMO1
BnaC03g45470D-F	5′- TCTTGCAACTATGATCCTCGGG -3′	PR1
BnaC03g45470D-R	5′- ACGTCCTATATGCACGTGTT -3′	PR1
PR4-1-F	5′- GCGGTAGATGCTTAAGAGTGAC -3′	PR4
PR4-1-R	5′- CCCGTTACTGCACTGATCCA -3′	PR4
PR4-2-F	5′- AGTGCTTAAGGGTGAGGAACA -3′	PR4
PR4-2-R	5′- ACATTGCAACGTCCAAATCCA -3′	PR4
